# Micronutrient Status, Health Implications, and Assessment Aproaches in Older Adults: A Narrative Review of Recent Studies

**DOI:** 10.3390/life16040570

**Published:** 2026-04-01

**Authors:** Hajnal Finta, Calin Avram, Corneliu-Florin Buicu, Daniela-Edith Ceana, Iuliu Moldovan, Florina Ruta

**Affiliations:** 1 Department of Public Health and Health Management, George Emil Palade University of Medicine, Pharmacy, Science and Technology of Targu Mures, Gheorghe Marinescu Street No. 38, 540139 Targu Mures, Romania; hajnal.finta@umfst.ro (H.F.); florin.buicu@umfst.ro (C.-F.B.); iuliu.moldovan@umfst.ro (I.M.); 2 Department of Medical Informatics and Biostatistics, George Emil Palade University of Medicine, Pharmacy, Science and Technology of Targu Mures, Gheorghe Marinescu Street No. 38, 540136 Targu Mures, Romania; 3 Department of Community Nutrition and Food Safety, George Emil Palade University of Medicine, Pharmacy, Science and Technology of Targu Mures, Gheorghe Marinescu Street No. 38, 540136 Targu Mures, Romania; florina.ruta@umfst.ro

**Keywords:** elderly, institutionalized elderly, older adults, vitamin D deficiency, malnutrition, zinc deficiency, vitamin B12, selenium, micronutrient

## Abstract

As populations age, micronutrient deficiencies increase and are linked to frailty, functional decline, cognitive impairment, anemia, and a higher healthcare burden. This review synthesizes evidence from the past five years on adults ≥65 years, comparing residents of nursing homes/assisted-living facilities with community-dwelling peers. Community-dwelling older adults show high prevalence of deficiencies—particularly vitamin D, calcium, magnesium, folate, and zinc—while vitamin B12 deficiency is less common overall but increases with age due to malabsorption. Institutionalized adults face higher risk, driven by limited dietary variety, reduced sunlight exposure, greater multimorbidity, and polypharmacy. Reported rates include vitamin D deficiency in 70–94% of institutionalized adults (≈6.3-fold higher odds), zinc deficiency in 50–66% (vs. 31–49% in the community), iodine deficiency in 67–78% (vs. 22% in the community), and a Mini Nutritional Assessment classification of severe malnutrition/at risk in 67.9% (vs. 28% in the community). Consequences encompass frailty, falls, infections, higher costs, and increased institutionalization. Recommended actions include routine biomarker screening, improving access to vitamin D (supplementation/fortification), individualized care for micronutrient deficiencies—including vitamin B12 when relevant—multidisciplinary nutrition support, and long-term targeted research to guide best practices for healthy aging and equity.

## 1. Introduction

The global population is aging at an unprecedented rate due to reduced fertility, longer life span, and better health. The global population aged 60 years and older was approximately one billion in 2020 and is projected to increase to 1.4 billion by 2030, when one in six people worldwide will be aged 60 or older, reaching 2.1 billion by 2050 [1]. The global share of the elderly population aged 60 and above is projected to rise to 22% in 2050, up from 12% in 2015, with four-fifths of the elderly population living in low- and middle-income nations by the middle of the century (World Health Organization (WHO)). The demographic transition increases challenges to population health, including the burden of chronic diseases, functional impairment, and higher healthcare costs, underscoring the need for targeted nutritional interventions to support healthy aging.

Vitamins and minerals enhance immune competence, bone mineralization, neuromuscular functions, cognitive functions, and hematopoiesis. In contrast, deficiencies promote sarcopenia, osteoporosis, anemia, cognitive impairment, and susceptibility to infections [2]. An example is vitamin D, which plays a role in absorbing calcium, making muscles stronger, and preventing falls; vitamin B12, which preserves neurological health and eliminates megaloblastic anemia; zinc, which supports immunity and wound healing; folate, which stimulates DNA growth and break down homocysteine (Feehan et al. 804). Poor intake and utilization are worsened by age factors, including low gastric acid secretion, poor absorption, polypharmacy, anorexia of aging, and long-term inflammation, which predisposes older adults to micronutrient deficiencies [2].

Examples of micronutrient inadequacies frequently reported in older adults include insufficient intake or status of vitamin D, vitamin B12, folate, zinc, iron, and magnesium. Vitamin D inadequacy is particularly common, with studies reporting prevalence estimates based on both dietary intake and biochemical status; among nursing home residents, biochemical deficiency can range from 8% to 94%, depending on cut-off values and assessment methods [3,4].

Evidence also indicates that suboptimal intake or biochemical status of vitamin B12, folate, zinc, iron, and magnesium may occur in older populations, although reported prevalence varies considerably depending on the population studied and the assessment method used (dietary intake versus biochemical markers) [5].

Institutionalized older adults are generally more susceptible to micronutrient inadequacies compared with community-dwelling peers due to several factors, including limited food choices, standardized menus with lower nutrient density, reduced sunlight exposure, higher levels of comorbidity, and dependence on staff for feeding. These conditions may contribute to an increased risk of nutritional deficiencies in long-term care settings [3].

Comparative research shows many significant differences. For instance, based on Mini Nutritional Assessment scores, 67.9% of persons over 65 years of age who were previously living in their community but became institutionalized after going to the hospital were identified as malnourished, compared to just 28% among those who remained in the community [6]. In nursing homes, rates of vitamin D deficiency, defined as plasma 25-hydroxyvitamin D levels < 50 nmol/L, have been reported to range from 8% to 94%. These wide ranges reflect heterogeneity due to geographic location, seasonal variation, population characteristics, and differing cut-off values used to define deficiency, which should be interpreted carefully rather than as a single estimate. Limited outdoor exposure, poor diet, and insufficient supplementation in institutional settings exacerbate these deficiencies, accelerating functional decline.

This review, based on data published over the past five years, will identify how age-related physical and environmental factors influences micronutrient status; identify assessment techniques; compile the prevalence of several major micronutrients; critically review older persons living at home or in institutions; and provide evidence on how their health may be affected, explain how they are affected, and provide recommendations for health-based interventions for equitable healthy aging. This review relies on recent sources and addresses data that have not been utilized in comparative analyses, thereby improving our understanding of how to screen, supplement, and take action through public policy to achieve equitable, healthy aging.

### 1.1. Literature Search Strategy

This narrative review aimed to synthesize recent evidence on micronutrient deficiencies in older adults, with a particular focus on differences between community-dwelling and institutionalized populations. A structured literature search was conducted using the electronic databases PubMed, Scopus, and Web of Science.

Search terms included combinations of micronutrient deficiency, vitamin D, vitamin B12, folate, zinc, magnesium, elderly, older adults, nursing home residents, institutionalized elderly, community-dwelling older adults, malnutrition, and nutritional assessment.

### 1.2. Inclusion and Exclusion Criteria

Studies were included if they had the following: involved adults aged ≥ 60 years; evaluated micronutrient status or dietary intake; included community-dwelling or institutionalized populations; were observational studies, systematic reviews, or cohort studies; and were published in English in peer-reviewed journals.

Studies were excluded if they had the following: focused on younger populations; examined disease-specific populations without relevance to aging; or lacked methodological description or primary data.

### 1.3. Study Selection and Data Synthesis

Titles and abstracts were screened for relevance, followed by full-text evaluation. Due to substantial heterogeneity in study design, population characteristics, micronutrient biomarkers, and diagnostic thresholds, a narrative synthesis approach was used rather than a quantitative meta-analysis.

### 1.4. Rationale for Narrative Review Design

A narrative review was selected because the available literature includes heterogeneous observational studies with differing methodologies, outcome definitions, and population characteristics. This approach allows for a broader conceptual synthesis of evidence and facilitates comparison between institutionalized and community-dwelling populations.

An initial search identified approximately 110 studies published between January 2021 and December 2025, along with selected earlier works to provide context. After applying the inclusion criteria, a total of 35 studies conducted across various geographic regions, including Europe, North America, and Asia, were included in the final analysis. Most focused on micronutrient deficiencies among older adults living in institutional settings such as nursing homes or long-term care facilities.

## 2. Age-Related Changes Impacting Micronutrient Status

Older adults experience a complex interplay of factors that compromise their ability to consume, absorb, and utilize essential nutrients, increasing the risk of micronutrient deficiencies [7]. This self-perpetuating cycle begins with age-related physiological changes, which weaken physical and metabolic functions.

Anorexia of Aging. Reduced appetite, often referred to as “anorexia of aging,” develops due to sensory deficits (impaired taste and smell), delayed gastric emptying, changes in gastrointestinal hormones (lower ghrelin, higher cholecystokinin and peptide YY), and the central effects of inflammation-related cytokines (e.g., TNF-α, IL-1β) on hypothalamic appetite centers [8]. These factors result in early satiety, reduced food intake, and insufficient consumption of vitamins and minerals.

Physiological Changes. Gastrointestinal function and nutrient bioavailability are particularly affected. Inadequate fluid intake, common among older adults, may further compromise absorption. Available evidence indicates that the physicochemical and microbiological differences between tap water and bottled water are not significant, suggesting that the volume of water consumed, rather than the source, is the key factor in maintaining homeostasis [9]. Vitamin B12 absorption can be limited by low gastric acid, reduced pepsin activity, and decreased intrinsic factor, affecting 20–40% of older adults, with up to 70% developing deficiencies [10]. Villous atrophy reduces the surface area for nutrient absorption, and age-related decreases in renal 1-alpha-hydroxylase reduce the conversion of vitamin D to its active form, resulting in lower calcium absorption and higher risk of vitamin D and calcium deficiencies [10]. B vitamins, vitamin D, and calcium are most severely affected.

Comorbidities and Polypharmacy. Chronic diseases are highly prevalent among older adults, particularly in nursing homes, leading to inflammation, altered metabolism, and increased nutrient requirements [2]. Multimorbidity is associated with lower 25(OH)D levels (odds ratio 1.28 per additional chronic disease) and an increased risk of anemia due to iron and other micronutrient sequestration. Polypharmacy can exacerbate deficiencies by reducing gastric acid (affecting vitamin B12, iron, magnesium, calcium absorption), causing gastrointestinal side effects (diarrhea, dry mouth), altering appetite, and affecting protein nutrition and taste perception [11]. Polypharmacy is strongly associated with malnutrition (adjusted OR 5.88, 95% CI 2.64–9.91) [11]. The use of opioids can reduce appetite due to nausea and constipation, while neuroleptic and procognitive drugs may negatively affect protein intake and taste perception.

Psychological and Social Factors. Social isolation, institutionalization, and depression reduce dietary variety, sunlight exposure, and overall nutrient intake [2]. Low physical activity further increases the risk of vitamin D deficiency (OR up to 6.20 for low vs. high activity) and accelerates frailty.

Overall Impact. The interaction of physiological changes, comorbidities, polypharmacy, anorexia of aging, and psychosocial factors leads to increased nutrient inadequacy, physical deterioration, frailty, disease risk, and loss of independence. These effects often go unrecognized until well-established, making timely nutritional assessments and targeted nutritional interventions essential for supporting healthy aging, including the appropriate use of specialized nutritional products when clinically indicated [12].

## 3. Assessment Methods for Micronutrient Status in Older Adults

Micronutrient status assessment among the elderly is essential for early detection of deficiencies, as the older population is vulnerable to deficiencies associated with health deterioration. Techniques include diet evaluation, biochemical analysis, clinical examination, and anthropometric analysis, which are often combined into standardized instruments such as the Mini Nutritional Assessment (MNA) [13]. A systematic review of dietary assessment in the elderly provides insights into the necessity of a customized approach. In contrast, general methods may not capture consumption due to cognitive issues or recall errors [14]. Self-reported instruments are feasible for community-based older adults. In contrast, institutionalized environments need to rely on proxy instruments, such as menu analyses. These approaches involves describing important approaches, their uses, advantages, weaknesses, and differences between populations.

## 4. Dietary Assessment Methods

Methods used to determine dietary intake involve quantifying food consumption to assess shortfalls in nutrient intake compared to the Estimated Average Requirement (EAR). Several dietary assessment methods are available, including the most common 24 h recall, food frequency questionnaire (FFQ), and weighed food record [15]. A systematic review of studies conducted with institutionalized older adults published in 2024 showed that weighed food records were utilized in 50% of studies [16]. In community settings, FFQs are more commonly used to assess long-term dietary patterns; however, under-reporting of intake is an issue for 30–50% of older adults due to difficulties with memory (Leij-Halfwerk et al.) [17].

Validated FFQs for vitamins D, B12, and zinc show a moderate level of reliability (correlation coefficients between 0.4 and 0.6) [18]. Portion sizes will need to be adjusted for the applicable population of frail individuals to ensure validity. The bulk of nutritional analysis occurs in long-term care (LTC) settings, with 29.4% of studies assessing menu nutrition as planned and consumed; however, no analysis is done for unit nutritional waste or resident preferences [16]. Digital tracking tools, including applications, are available for the elderly in the community and show increased accuracy from 15 to 20% in pilot test results but have not been widely adopted in LTC settings due to potential technological limitations [19].

## 5. Biochemical Assessment

Using blood, urine, or tissue samples, biochemical markers objectively assess status. In the case of vitamin D, 25-hydroxyvitamin D (25(OH)D) is the gold standard and is considered to be low (<50 nmol/L) when deficient. However, because of the high levels of acute-phase proteins resulting from the presence of inflammatory diseases in multimorbid older adults, values may be spurious [20]. A combination of serum vitamin B12 and functional testing through methylmalonic acid is used to estimate malabsorption, which affects 20–40% of these individuals [21]. Selenium and zinc levels are typically measured in blood via plasma analysis, but deficiencies are often masked by homeostasis; hair or nail testing shows long-term status but it is not standardized for older adult populations [2]. A 2023 review suggested performing routine panels in at-risk populations, specifically identifying that testing at outpatient (community settings) laboratories is more feasible than at institutional (due to logistical limitations like venipuncture of a bedbound person) settings [3]. Cut-off values should likely be recalibrated to account for age-related renal functioning decline, which has been demonstrated to affect thresholds.

### Nutritional Screening and Assessment Tools

Nutritional screening and assessment tools are essential for identifying older adults at risk of malnutrition. The Malnutrition Universal Screening Tool (MUST) is appropriate for community-dwelling adults, showing high specificity (85%). In contrast, the Nutritional Risk Screening 2002 (NRS-2002) demonstrates lower sensitivity [22]. The Patient-Generated Subjective Global Assessment (PG-SGA) incorporates symptom-based scoring, which is particularly useful in multimorbid patients [23] (“Patient-Generated Subjective Global Assessment (PG-SGA) Tool to Evaluate the Nutritional Status of Patients—CCI4EU Resource Centre”).

**Clinical Methods.** Clinical manifestations can identify obvious deficiencies, such as angular stomatitis in vitamin B deficiency or night blindness in vitamin A deficiency. However, in older adults, these signs often appear late and may overlap with comorbidities.

**Anthropometric Methods.** Anthropometric measures provide objective data: a body mass index (BMI) below 18.5 kg/m^2^ indicates undernutrition, while calf circumference or bioimpedance analysis (BIA) can indicate sarcopenia. BIA measures body composition but is less precise in dehydrated elderly [24].

**Integrated Assessment.** Using clinical, anthropometric, and dietary history methods together improves reliability in determining malnutrition [25]. Combining these approaches ensures a more accurate representation of an individual’s overall nutritional risk than relying on a single method alone.

## 6. Disparities and Gaps

Community-based elderly individuals benefit from ambulatory evaluation processes: they would receive regular blood screening and FFQ, although access is not consistently effective (a significant percentage remain unscreened in LMICs). Institutionalized individuals have an advantage in detecting signs and symptoms of malnutrition through proxy measures (such as menu records); however, differences between these methods create inconsistencies across studies, limiting the replicability of their findings [26]. Variability in testing practices between institutional and community settings may also contribute to detection bias when comparing prevalence estimates. Gaps include the use of digital technologies (preferably real-time monitoring); the ability to validate supercentenarians will also need to change due to more questionable data [27]. Future experimental designs will need to utilize a standardized protocol and artificial intelligence (AI) to limit bias.

## 7. Prevalence and Patterns of Micronutrient Deficiencies in the Elderly

### Sources of Heterogeneity in Prevalence Estimates

Reported prevalence of micronutrient deficiencies varies widely across studies due to methodological and population differences. Geographic location influences dietary patterns, sunlight exposure, and healthcare access. Additionally, studies use different biochemical cut-off values to define deficiency (for example, vitamin D deficiency thresholds ranging from 25 nmol/L to 50 nmol/L). Seasonal variation also affects vitamin D status, while population characteristics such as age distribution, frailty status, multimorbidity, and institutionalization influence nutritional risk. These factors should be considered when interpreting prevalence estimates across studies.

Older adults commonly experience micronutrient deficiencies, influenced by where they live, dietary intake, absorption efficiency, and comorbidities. Several systematic reviews and cohort studies indicate that micronutrient deficiencies among institutionalized older adults are generally more severe than among community-dwelling older adults, primarily due to restricted diets, limited physical mobility, and minimal exposure to sunlight. This section will summarize data on the prevalence of various key micronutrients using both recent studies published between 2021 and 2025 and earlier high-quality studies, and identify differences between community-based and institutional-based older adults.

However, these differences should be interpreted with caution, as micronutrient levels may be assessed more frequently in institutionalized settings than in community populations.

## 8. Vitamin D

Among the many factors contributing to vitamin D deficiency in older American adults are inadequate dietary intake, limited sun exposure, and impaired renal activation. Among community-dwelling elderly worldwide, the prevalence of vitamin D deficiency (values less than 20 ng/mL or 50 nmol/L) is 59.7% [28]. It is important to clarify that these prevalence estimates include both biochemical deficiency (measured via serum 25[OH]D) and reported dietary inadequacy; differences in method contribute to variability. The prevalence of vitamin D deficiency among older adults varies by region, with an overall rate of approximately 20% and disproportionately higher rates observed among non-White ethnic groups and individuals of lower socioeconomic status [29] (Table 1).

A systematic review found a greater deficiency is present in institutionalized older adults with prevalence rates ranging from 8% (less than 25 nmol/L) to 94% (less than 50 nmol/L), reflecting heterogeneity from the different cut-offs used, seasonal variation, sunlight exposure, and health status, which may be two to three times higher than those who live in the community due to limited mobility, low levels of exposure to sunlight, and inadequate dietary intakes [3]. The odds of being deficient were much greater among nursing home residents and were associated with measures of frailty and risk of falling [3]. Seasonal fluctuations also compound this problem, as lower levels are found throughout the winter months.

## 9. B-Group Vitamins (B12, Folate)

Vitamin B12 or folate levels drop due to inadequate nutrition, not eating enough food, or taking too many medications. Older adults living at home and having low vitamin B12 (less than 148 pmol/L) are present in 17.2% of that age range (60–85). They have a very low risk of low folate levels (less than 10 nmol/L), with only 1% of this population group affected. It should be clarified that the previously reported rates of “50–88% suboptimal intake” refer to dietary intake estimates, not confirmed biochemical deficiency. Also, individuals who are deficient or have low B12 levels have an increased risk of developing a new episode of depression by 51% over four years.

Older adults living in a long-term care setting are at much greater risk of vitamin B12 deficiency (34% based on biochemical measurements) than younger controls (9%). They are also at an increased risk of folic acid deficiency. The approximately two to three times greater deficiency in institutions reflects both dietary inadequacy and impaired absorption. For example, the rate in institutionalized groups is approximately two to three times greater than that in the community, with an estimated 70% of deficiencies related to malabsorption [11] (Table 1). Additionally, rates of anemia are greater in institutional settings; 67–93% of residents in long-term care settings are estimated to be at risk (as determined by the Mini Nutritional Assessment) versus only 28–70% in the community.

## 10. Vitamins C and E

Vitamins C and E are antioxidants that help guard against oxidative stress, but inadequacies are common due to low fruit/vegetable intake. Among community-dwelling older adults aged 60 years and older, vitamin C deficiency is prevalent among a substantial proportion of the population [30] (Table 1). It should be clarified that reported prevalence estimates vary depending on whether deficiency is defined using dietary intake assessments or biochemical plasma vitamin C concentrations, as these methods may yield different results.

The situation is worse with institutionalized elderly, where vitamin C inadequacy is 40% compared to free-living elderly [30]. This estimate primarily reflects inadequate dietary intake rather than confirmed biochemical deficiency, and differences across studies may arise from variation in menu composition, supplementation practices, and assessment methodology. Comparative analyses indicate that plasma levels are very low in institutions compared to the community, and deficiency rates are 2–3 times higher due to monotonous diets and illness [30] (Table 1). However, these comparisons should be interpreted cautiously, as institutionalized populations often present higher multimorbidity, inflammation, and functional dependency, which may independently influence circulating vitamin C levels. There is no total deficiency, but a borderline deficiency is common, which makes one prone to infections. Borderline deficiency thresholds also differ between studies, contributing to heterogeneity in reported prevalence.

## 11. Minerals (Iron, Zinc, Calcium, Magnesium, Selenium)

Mineral deficiencies contribute to anemia, immune deficiencies, and skeletal frailty. Among older people living independently, there is geographic variation in the prevalence of iron deficiency (ferritin < 30 ug/L) in high-income countries 2–4.6%, and 49.8% in some regions using transferrin receptor–ferritin indices [2]. These variations reflect differences in methodology, population iron status, dietary patterns, and assessment criteria. Zinc deficiency ranges are as follows: 28–42% in rural areas; biochemical results: 31% of women and 49% of men [2]. High levels of inadequacy are found for calcium and magnesium, with 65–73% and 41% to 73%, respectively, below the EAR due to dairy avoidance or absorption issues [2]. Selenium deficiency is also high (37% to 49%) among older community-dwelling adults (Table 1).

Deficiencies are compounded in an institutional setting. The prevalence of zinc deficiency may be 50–66%, iron deficiency to 31%, and selenium deficiency to 27–44%. In some nursing home groups, the prevalence of calcium and magnesium inadequacy exceeds 90%, and fortified menus often fall short [2]. These percentages reflect both dietary inadequacy and functional absorption issues in older adults, which should be explicitly noted. The overall prevalence of deficiencies in institutions is 1.5 to 2 times higher than in the community, thus contributing to the frailty cycle (Table 1).

These trends underscore the importance of specific actions, as the absence of certain factors exacerbates health degradation. The elderly living in communities enjoy a variety of diets, whereas institutionalized elderly need supplementation and menu fortification. Future studies will need to use longitudinal data to improve cut-offs and consider regional differences.

## 12. Comparative Analysis: Institutionalized vs. Community-Dwelling Elderly

Older adults’ nutrition-related vulnerabilities vary widely depending on their housing situation. Institutionalized older adults (like those living in nursing homes or long-term care facilities) often have an increased risk of having a poor diet relative to older adults living in the community.

Gaps have been noted in cross-sectional and comparative research from 2021 through 2026, often using assessment tools such as the Mini Nutrition Assessment (MNA) to determine general malnutrition risk and biochemical analysis to measure specific nutrients. The evidence indicates higher deficiencies for those living in institutional settings due to lifestyle restrictions compared with those living in the community; however, both groups have increased malnutrition risk, primarily due to the aging process [31,32]. In addition, differences in the frequency of biochemical testing between institutional and community settings may contribute to detection bias and influence the reported prevalence of deficiencies. The purpose of this review was to synthesize recent observational evidence to compare general malnutrition and micronutrient profiles and to highlight potential contributory factors.

## 13. General Malnutrition Risk

Older people experience malnutrition (undernutrition or inadequacies of nutrients), which increases their chances of becoming frail, being hospitalized, and mortality. The Mini Nutritional Assessment (MNA) tool will help classify older people as either standard (≥24 points), at risk (17–23.5 points), or malnourished (<17 points). When comparing various studies, older people living in institutions will have a much higher prevalence of malnutrition because they are dependent on others for their needs and live in an environment that is constrained with respect to food choices.

A previous study evaluated the nutrition of seniors using the MNA Scale: 125 community-dwelling vs. 125 institutionalized females aged ≥ 60 years. Results show that 30.4% of community-dwelling individuals had normal scores. In comparison, 69.6% had an increased chance of malnutrition (mean MNA: 23), whereas only 7.2% of institutionalized individuals had normal scores, while 92.8% had an increased chance of malnutrition (median MNA: 19). The difference was statistically significant (Mann–Whitney test, *p* < 0.05); however, in terms of malnutrition, the odds ratio for institutionalized versus community members was 0.50 (95% CI: 0.10–2.33; not significant) [33]. A 2025 preprint review cited data on malnutrition in the U.S.; for example, malnutrition among community elderly ranged from 1 to 15% versus 25 to 85% for those in nursing homes, while malnutrition rates for older adults living in community settings in Taiwan were approximately 3.6% versus 20.7% for those living in nursing homes [34] (Table 2).

A 2025 cross-sectional analysis of 102 nursing home residents (mean age = 87.8) reported that 47.1% were at high risk for malnutrition, while 13.7% were malnourished based on the MNA-Short Form (MNA-SF average = 10.24). Decreased MNA-SF scores were correlated with difficulty eating; 65% of participants needed assistance with meals. For the total population studied, there was a statistically significant negative correlation between MNA score and difficulty eating (r = −0.63, *p* < 0.001) (Table 1) [33,35]. Comparatively, the community has a lower malnutrition risk based on a systematic review of 2022 studies regarding Indonesian community-dwelling adults, where malnutrition ranged from 2.1% to 14.6% and being at risk (for malnutrition) ranged from 18% to 78% (Table 1) [33,35,36]. Data show that the global age-standardized prevalence rates (ASPR) for protein–energy malnutrition (PEM) in adults 65 years of age and older increased from 1407.16 to 2015.58 per 100,000 between 1990 and 2021. The highest rates of prevalence were found in low-income geographic areas, which have implications regarding the association with increased risk of PEM in institutionalized older adults living in resource-restricted settings (Table 2).

These results highlight a vicious cycle: malnutrition is a factor that leads to institutionalization, while institutional conditions aggravate it. There is a dearth of longitudinal data, but cross-sectional evidence indicates a 2- to 3-fold increased risk in institutions.

## 14. Specific Micronutrient Comparisons

Institutionalized older adults are at a higher risk of severe micronutrient deficiencies than their community-dwelling peers [37]. This increased risk results from several factors, including inadequate food intake, poor nutrient absorption, and the environments in which institutionalized elderly persons live. These include limited food variety, the use of standard menus, low nutrient density of the food served, limited ability to select food, very little exposure to sunlight, and a need for assistance with eating. Physiological changes associated with aging will also contribute to some risk, as do many chronic medical conditions and chronic inflammation. Additionally, the widespread use of certain medications, such as proton pump inhibitors, metformin, and diuretics, can complicate or limit the bioavailability of specific nutrients, including vitamin D, vitamin B12, iron, zinc, and magnesium. Data derived from systematic reviews and cohort studies show that there are far higher incidence rates of deficiency in institutional environments than in community environments [38]. The tabulated synthesis of the results of these studies allows for a side-by-side comparison of prevalence across living environments, providing opportunities to see the extent of the disparity between deficiencies in the nutritional status of institutional residents and community-dwelling older adults. Such comparative data support the evidence that an institutional environment increases the risk of nutritional vulnerability, and the necessary nutrition-specific to the environment must be implemented; specific screening, fortification, and individual supplementation should be performed to address the health outcomes of deficiency among long-term care populations.

Vitamin D deficiency is emblematic: A 2022 systematic review by Feehan et al. [3] found that between 8% (<25 nmol/L) and 94% (<50 nmol/L) of nursing home residents had a deficiency or were not supplemented [3] (Table 3). It is critical to clarify that the wide range reflects different biochemical cut-offs, seasonal variation, and varying assessment methods, rather than contradictory findings. Brîndușe et al. have shown that populations of older adults living outside of nursing homes had significantly lower percentages (40–60%) than those identified in nursing homes (804) [39]. Malnourished elderly hospitalized patients had vitamin D deficiency, indicating similar levels of deficiency as those found among institutionalized older adults.

B vitamin inadequacy is common in community settings and increases further among institutionalized populations. The probability of zinc deficiency is 12–49% in the community and 50–66% in institutions. According to a 2025 multicenter study of nursing homes, over 90% of respondents had inadequate intakes of vitamin D, magnesium, and potassium, and poor nutritional status despite 63% of respondents being supplemented [2], indicating dietary insufficiency; however, biochemical confirmation may vary. Data from the global GBD reveal that ASPR iodine (1921.43 to 1656.34) and vitamin A (6538.09 to 2362.40) decrease, while PEM increases, and iron deficiency remains at the same rate (just under 14,845.71 per 100,000) [40].

Multiple deficiencies (>3) were found in malnourished elderly patients in a hospitalized setting, including vitamin C, biotin, and selenium [41]. The comparative evidence posted in 2023–2025 shows a higher prevalence in institutions, 1.5–3 times higher than in the general population.

## 15. Contributing Factors

### Several Factors Contribute to Micronutrient Deficiencies in Older Adults

Dietary Quality: Institutionalized individuals often consume repetitive, nutritionally poor meals, with over 90% failing to meet micronutrient requirements. Community-dwelling older adults generally have access to a wider variety of foods, though age-related anorexia can reduce intake [9]. A 2023 study highlighted low micronutrient intake among nursing home residents and recommended a food-first approach [42]. Beyond dietary intake, assessing these deficiencies in real-world settings can be challenging. Practical approaches, including routine dietary screening, laboratory monitoring, and targeted supplementation, are essential. Healthcare professionals should actively identify and correct nutritional deficiencies to improve health outcomes in both institutionalized and community-dwelling older adults (Table 4).

Sun Exposure: Limited outdoor time, especially in residential care facilities, increases the risk of vitamin D insufficiency (≈6.3-fold higher odds than in the community) [3]. Active community elders show better vitamin D status (odds ratio 6.20 for low vs. high activity) [2] (Table 4).

Care Environment: Dependence on feeding assistance in institutions raises malnutrition risk; 65% of residents require help, with higher care needs correlating with lower MNA scores [35]. Lack of family support at home also increases the risk of depression and malnutrition [33], while longer institutional stays negatively impact nutritional status (r = −0.292, *p* = 0.037) [35] (Table 4).

Functional Status: Cognitive impairment is inversely related to nutritional status (MoCA average 14; r = 0.401, *p* = 0.037) [30], and malnutrition is associated with depression (*p* < 0.0001) [33]. Multimorbidity and polypharmacy further increase deficiency risk, particularly among those with higher care needs (Table 4).

## 16. Evidence from Cross-Sectional and Comparative Studies

Cross-sectional data predominate in the literature, with Shakil et al.’s [33] sample (N = 250) providing direct comparisons showing that institutional factors, such as illiteracy and being single, increase the risk of institutional placement [30]. Multicenter studies, such as those by Krušič et al., also report widespread inadequacies in long-term care. The Global GBD shows how pandemics increase the number of people in institutions (AAPC 1.18) [40,42]. Institutions and how they care for individuals with disabilities have changed dramatically since 2019. Regionally, comparing cohorts like those in Chen et al. suggest that a longitudinal clinical trial design that tracks individuals as they transition from their communities into institutions is needed [34].

Such inequalities require specific actions: enriched diets, regular screening, and individual fortification in institutions, whereas diverse diets and exercise should be encouraged at the community level. The holistic approach to these factors would help to reduce the decline in health and establish equity in aging.

## 17. Health Implications, Contributing Factors, and Recommendations

There is a strong relationship between frailty and inadequate intake of vitamin D, zinc, and magnesium. Vitamin D deficiency, defined by plasma 25-hydroxyvitamin D < 50 nmol/L, in elderly nursing home residents is associated with increased frailty based on the Fried phenotype score and a higher risk of falls, due to impaired muscle protein synthesis and reduced calcium absorption [3].

### Health Implications, Contributing Factors, and Recommendations

**Figure 1:**
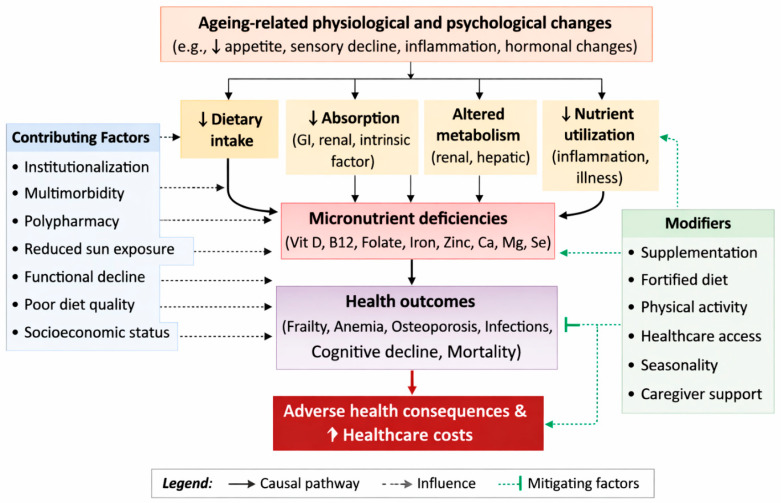
Conceptual framework of determinants of micronutrient deficiencies in older adults.

Older adults (≥65 years) are particularly vulnerable to micronutrient deficiencies, which can exacerbate age-related decline and increase the risk of frailty, anemia, cognitive impairment, functional decline, and higher healthcare burden. Where prevalence ranges are wide (e.g., vitamin D 8–94%), the heterogeneity is due to differences in biochemical cut-offs, sunlight exposure, geographic region, and institutionalization, which should guide interpretation and targeted interventions. Frailty is characterized by reduced strength, endurance, and physiologic function, and is closely associated with inadequate intake of vitamin D, zinc, and magnesium. Vitamin D deficiency, defined by plasma 25-hydroxyvitamin D <50 nmol/L, in elderly nursing home residents is associated with increased frailty based on the Fried phenotype score and a higher risk of falls, due to impaired muscle protein synthesis and reduced calcium absorption [3]. Zinc deficiency, affecting over half of institutionalized elders, compromises immune function, delays wound healing, and accelerates frailty and infection risk. Multimorbidity further increases adverse outcomes, while deficiencies in B vitamins and iron contribute to sarcopenia and reduced grip strength in community-dwelling older adults (Figure 1).

Anemia is common in older adults and is often due to deficiencies in iron, vitamin B12, or folate, leading to fatigue, reduced oxygen transport, and impaired functioning. Vitamin B12 insufficiency can cause megaloblastic anemia, neuropathy, and mobility limitations, while low folate or B12 elevates homocysteine, increasing dementia risk. Rising rates of protein–energy malnutrition intensify cognitive decline, disability, and healthcare costs, emphasizing the urgent need for targeted nutritional interventions.

**Contributing factors** include physiological changes such as hypochlorhydria (reducing B12 absorption), chronic conditions like diabetes (increasing inflammation and nutrient needs), and polypharmacy (e.g., PPIs reducing magnesium absorption; malnutrition odds ratio 5.88) [2,8]. Institutionalized elders face additional challenges, including limited dietary variety, reduced sun exposure (vitamin D deficiency odds ratio 6.3 vs. community), and dependence on caregivers for feeding [9]. Community-dwelling older adults, though more autonomous, may experience social isolation and anorexia of aging, decreasing their intake of nutrient-rich foods (Figure 1).

**Recommendations** include systematic screening of at-risk populations using biomarkers (25[OH]D, vitamin B12, ferritin) and validated nutritional assessment tools, routine individualized supplementation (year-round vitamin D for institutionalized adults; B12 for malabsorption; multivitamins/minerals for community-dwelling older adults), and implementation of nutrition programs to ensure menu fortification, nutrient-dense foods, and diet variety. Multidisciplinary teams should coordinate interventions to optimize intake, address barriers, and promote adherence. Policy support and ongoing research are essential to close persistent gaps and ensure equitable nutritional care for older adults. There are evidence-based guidelines for preventing nutritional deficiencies in older adults. Early gut dysbiosis raspunde has been linked to altered nutrient absorption and metabolic changes [43]. Early identification through annual screening of at-risk populations using biomarkers such as 25(OH)D, vitamin B12, and ferritin can help guide timely interventions [44]. Individualized supplementation should be provided for institutionalized older adults (e.g., year-round vitamin D), for those with B12 deficiency due to malabsorption, and for community-dwelling individuals when necessary (e.g., multivitamins/minerals). Nutrition programs should aim to fortify menus, produce nutrient-dense foods through multidisciplinary collaboration, and provide variety-focused initiatives to enhance micronutrient intake in the community. Persistent gaps in policy, research, and implementation highlight the need for ongoing, equitable, and evidence-based solutions to support healthy aging.

## 18. Limitations of the Study

A potential limitation of this review is the predominance of studies using the Mini Nutritional Assessment (MNA) as the primary screening tool. Although MNA is widely validated for older adults, other nutritional assessment instruments are also available, and their limited representation in the included studies may introduce a degree of selection bias.

## 19. Conclusions

Deficits of micronutrients such as vitamin D, B12, and zinc, as well as deficiencies in other vitamins and minerals, can lead to a range of problems, including accelerated frailty, anemia, cognitive dysfunction, and limitations in daily activities. These issues create a cycle of declining health and increased institutionalization. The results also highlight significant differences between community-dwelling and institutionalized older adults, emphasizing the urgent need for equitable interventions, such as routine screening, personalized supplementation (e.g., higher-dose vitamin D for institutionalized individuals), and fortified menus to reduce nutritional risks.

To support global efforts for nutrient-secure aging, longitudinal research is needed to monitor deficiency progression and evaluate intervention effectiveness, especially in lower-middle-income countries where protein–energy malnutrition remains prevalent. At the policy level, addressing food security, sustainable food systems, and community programs is essential to build resilience among older adults and promote equitable, nutrient-secure aging worldwide.

## Figures and Tables

**Table 1:** Common micronutrient deficiencies in older adults and associated risk factors.

Micronutrient	Foods	Physiological Role	Risk Factors in Older Adults	Potential Health Consequences
Vitamin D [28,29]	Fatty fish, fortified dairy, and eggs	Bone metabolism and immune regulation	Reduced sun exposure, low dietary intake, and institutionalization	Osteoporosis, fractures, and impaired immunity
Vitamin B12 [11]	Meat, fish, eggs, and dairy	DNA synthesis and neurological function	Reduced gastric acid, malabsorption, and inadequate intake	Cognitive decline, anemia, and neuropathy
Folate [11]	Spinach, kale, broccoli, lentils, chickpeas, citrus fruits, eggs, and fortified cereals	DNA synthesis and cell division	Poor dietary intake and chronic disease	Megaloblastic anemia and cardiovascular risk
Vitamin C [30]	Citrus fruits, strawberries, bell peppers, and broccoli	Antioxidant, collagen synthesis, and immune support	Low dietary intake, smoking, and chronic illness	Impaired wound healing, weakened immunity, and increased oxidative stress
Vitamin E [30]	Nuts, seeds, vegetable oils, and spinach	Antioxidant, protects cell membranes, and supports immune function	Low dietary intake, fat malabsorption, and chronic disease	Increased oxidative stress, cognitive decline, and cardiovascular issues
Iron [2]	Red meat, legumes, and spinach	Oxygen transport and energy metabolism	Chronic disease, reduced absorption, and poor diet	Iron deficiency anemia and fatigue
Zinc [2]	Meat, nuts, seeds, and whole grains	Immune function and wound healing	Reduced intake and chronic inflammation	Impaired immunity and delayed healing
Magnesium [2]	Nuts, seeds, green leafy vegetables, and whole grains	Neuromuscular function and metabolic processes	Medication use and reduced intake	Muscle weakness and metabolic disturbances

**Table 2:** Malnutrition risk by living setting.

Malnutrition Risk Category	Community-Dwelling (%)	Institutionalized (%)
Normal (MNA ≥ 24) [34]	30.4–80	7.2–52.9
At Risk (MNA 17–23.5) [33,35]	18–69.6	47.1–78
Malnourished (MNA < 17) [33,35,36]	2.1–14.6	13.7–85

**Table 3:** Micronutrient deficiency by living setting.

Micronutrient	Community Inadequacy/Deficiency (%)	Institutional Inadequacy/Deficiency (%)	Key Contributing Factors
Vitamin D [28,29]	40–60 (<50 nmol/L)	65–94 (<50 nmol/L)	Limited sunlight exposure, reduced mobility
Vitamin B12 [11]	16–19	34–70 (malabsorption-linked)	Malabsorption, medications
Folate [11]	29–35	37	Low dietary intake
Zinc [2]	12–49	36–66	Poor dietary diversity
Iron [2]	2–4.6 (high-resource)	31	Chronic inflammation
Magnesium [2]	41–73	>90 (inadequate intake)	Low dietary intake
Selenium [2]	37–49	27–44	
Vitamin C [27]	7–29	75	

**Table 4:** Factors contributing to micronutrient deficiencies in institutionalized older adults.

Category	Specific Factors	Impact on Nutritional Status
Dietary factors [9,42]	Standardized menus, low nutrient density foods	Inadequate intake of vitamins and minerals
Sun Exposure [2,3]	Reduced outdoor time, institutionalization, seasonal variation	Decreased endogenous vitamin D synthesis, increased risk of vitamin D deficiency
Care Environment [35]	Limited sun exposure, restricted mobility	Increased risk of vitamin D deficiency
Functional status [33]	Multimorbidity, chronic inflammation, Dysphagia, chewing difficulties, Dependence on staff for feeding	Increased nutrient requirements Reduced food intake Irregular intake and inadequate dietary support

## Data Availability

No new data were created or analyzed in this study.
